# Supplementary material for: The Staphylococcus aureus cell division protein, DivIC, interacts with the cell wall and controls its biosynthesis
Source: Commun Biol. 2022 Nov 11;5:1228. doi: 10.1038/s42003-022-04161-7 (PMC9652317; doi:10.1038/s42003-022-04161-7)
Supplement: Supplementary file 2 — Supplementary Information [file 42003_2022_4161_MOESM2_ESM.pdf]

Supplementary Information

The *Staphylococcus aureus* cell division protein, DivIC, interacts with the cell wall and controls its biosynthesis

Mariana Tinajero-Trejo[1,2#], Oliver Carnell[1,2#], Azhar F Kabli[1,2], Laia Pasquina-Lemonche[2,3], Lucia Lafage[1,2], Aidong Han[4], Jamie K Hobbs[2,3] and Simon J Foster[1,2*]

[1] School of Biosciences, University of Sheffield, Sheffield, UK.

[2] The Florey Institute for Host-Pathogen Interactions, University of Sheffield, Sheffield, UK

[3] Department of Physics and Astronomy, University of Sheffield, Sheffield, UK

[4] State Key Laboratory of Cellular Stress Biology, School of Life Sciences, Xiamen University, Xiamen, China.

[#] These authors contributed equally

* To whom correspondence should be sent. E-mail: s.foster@sheffield.ac.uk.

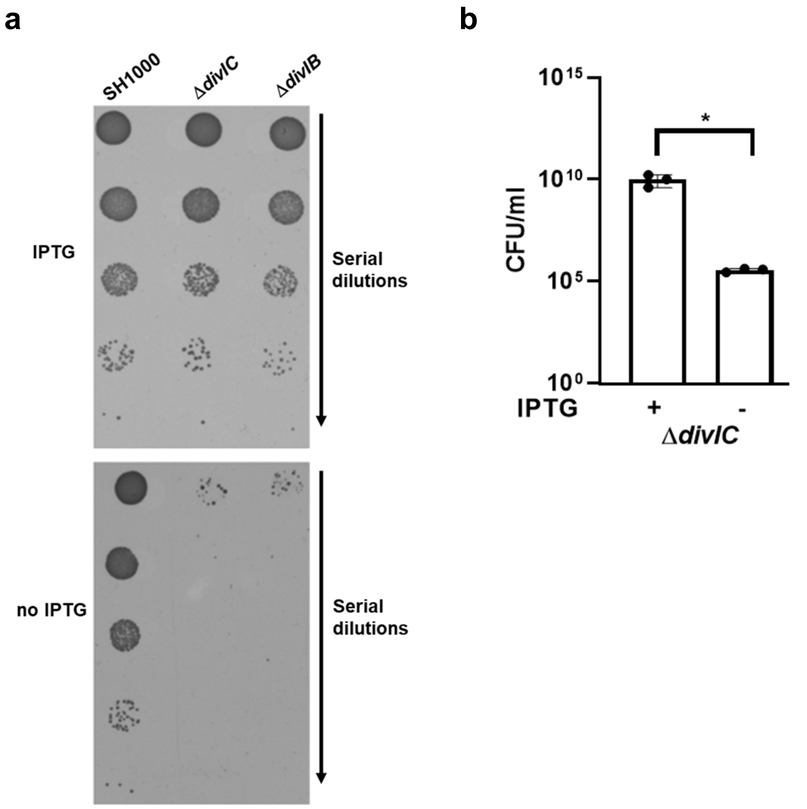

Supplementary figure 1. Loss of viability in the absence of DivIC and DivIB. **a,** Overnight growth of SH1000, Δ*divIC* (SJF5450) and a Δ*divIB* (SJF3883) strains in the presence and absence of IPTG. Figure is representative of two independent experiments. **b,** Viability test of Δ*divIC* (as in (a)) in the presence and absence of IPTG. Data represents the mean and standard deviation of three independent experiments. *P* value was determined by two-tailed unpaired *t* test (* *P* <0.0477).

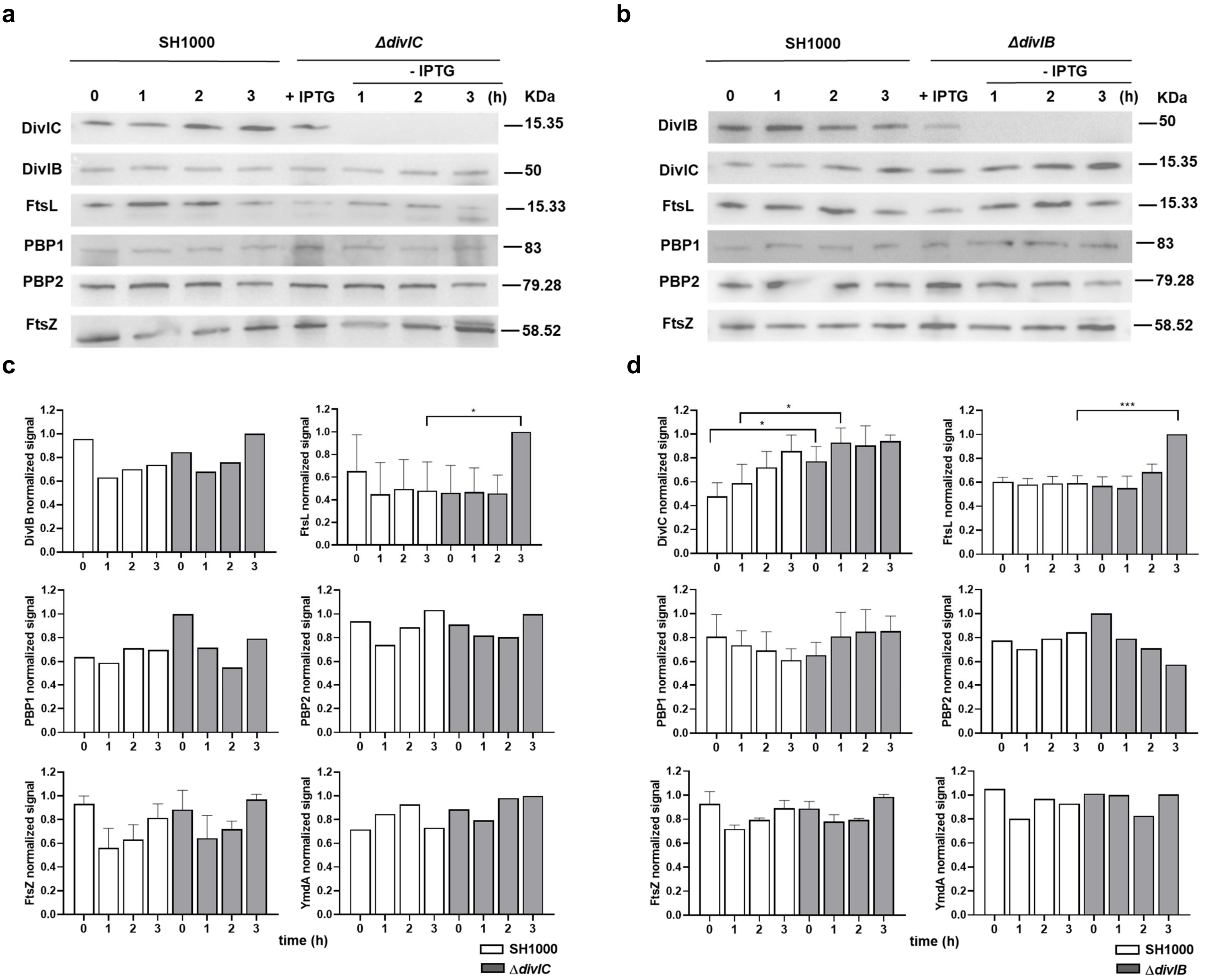

Supplementary figure 2. Role of DivIB and DivIC in protein stability. a, Western blot of whole cell lysates from SH1000, Δ*divIC* (SJF5450) and **b,** Δ*divIB* (SJF3883) strains grown with or without IPTG for 0, 1, 2 and 3 h. Anti DivIC, DivIB, FtsL, PBP1, PBP2, FtsZ antibodies were used for detection. YmdA antibodies were used as a control. Signal values from western blots were normalized against the integrated density of the signal from SDS-PAGE gels loaded with the same amount of protein and stained with Coomassie blue. **c** and **d** panels show values from (a) and (b) respectively. Average and standard deviations from three independent repeats are shown. Graph lacking statistics are representative of two independent repeats. *P* values were determined by two-tailed *t* test. In (c), **P*= 0.238. In (d), top-left graph, **P* =0.0387 (left) and **P* =0.0423 (right). Top-right graph, ****P* =0.0003.

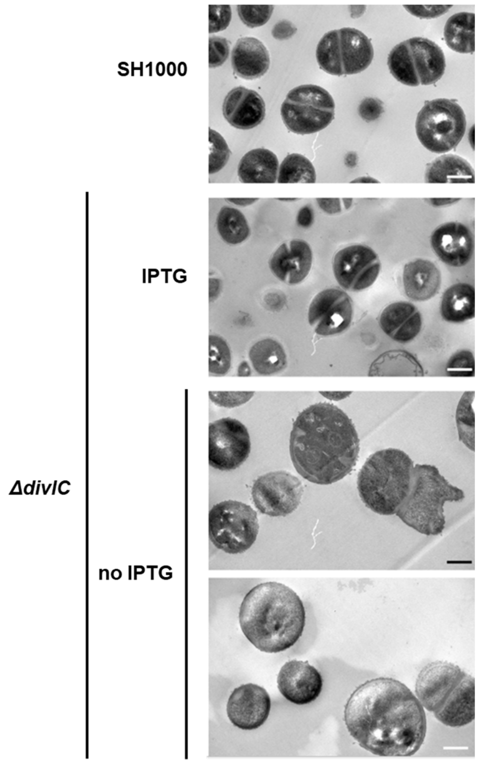

Supplementary figure 3. Use of TEM to analyse the role of DivIC in cell morphology. TEM of SH1000 and Δ*divIC* (SJF5450) grown for 3h in the presence or absence of IPTG. Magnification 4800X. Scale bar 0.5 µm. Images are representative of three independent repeats.

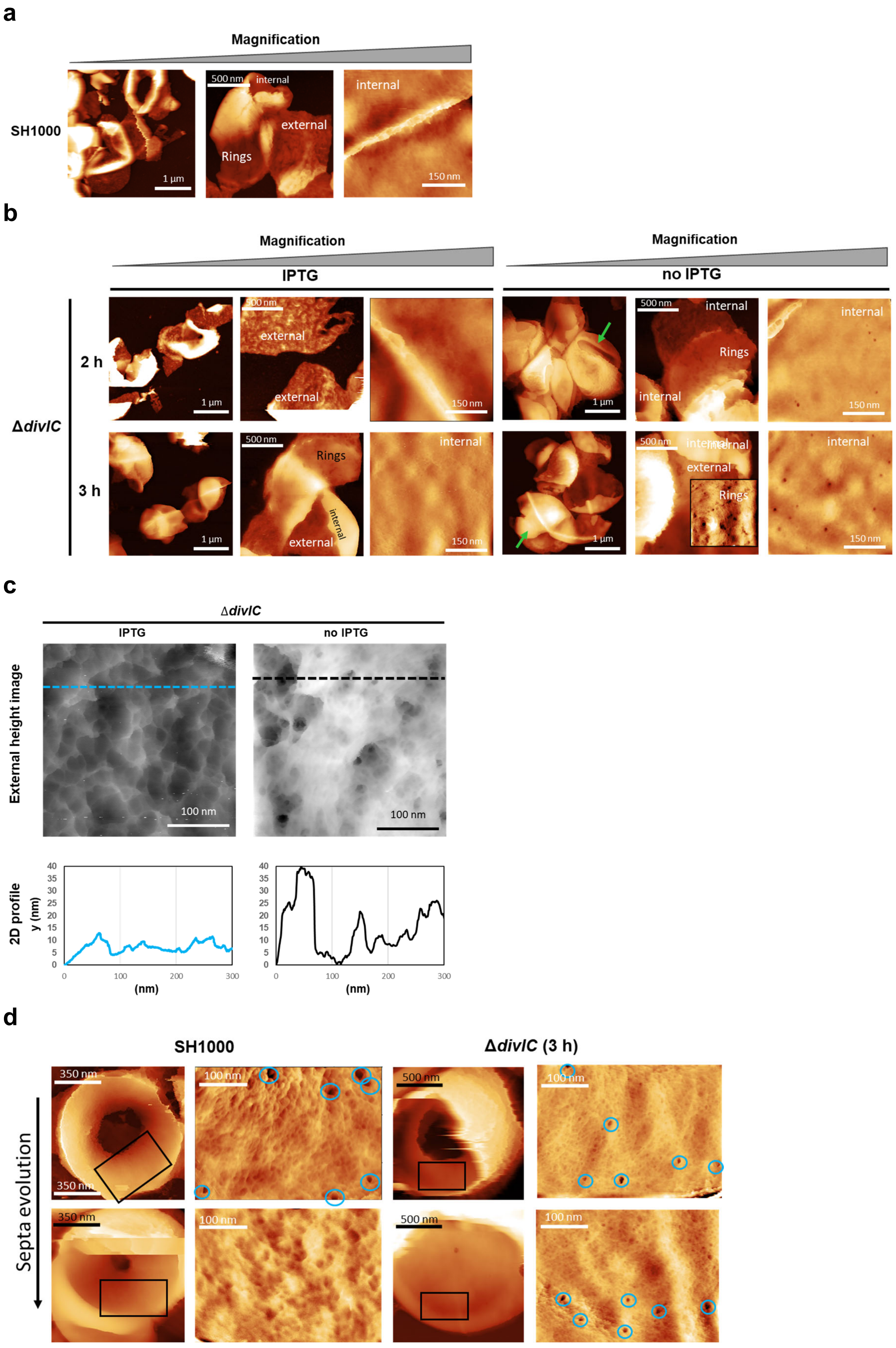
a
Magnification
SH1000
1 µm
500 nm
internal
external
Rings
internal
150 nm
b
Magnification
IPTG
Magnification
no IPTG
ΔdivIC
2 h
3 h
1 µm
500 nm
external
external
150 nm
internal
Rings
internal
Rings
internal
external
internal
external
Rings
100 nm
c
ΔdivIC
IPTG
no IPTG
External height image
2D profile
y (nm)
(nm)
d
SH1000
ΔdivIC (3 h)
Septa evolution
350 nm
100 nm
500 nm

Supplementary figure 4. Use of AFM to analyse cell wall architecture. a, AFM images in liquid in PeakForce mode. **a,** General morphology overview of cell walls from a mixture of SH1000 cells grown to mid exponential phase (2 and 3h). **b,** General morphology overview of cell walls from Δ*divIC* (SJF5450) grown for 2 and 3h in the presence or absence of IPTG. The same sequential increase in magnification used in panels (a) and (b) for comparison, as shown by scale bars. Green arrows highlight enlarged sacculi after growth in the absence of IPTG. **c,** Features of the cell wall external surface of Δ*divIC* (SJF5450) grown for 3h in the presence and absence of IPTG (top). 2D line profiles (bottom) showing the thickening of the PG layers in the absence of IPTG and a rougher external surface with bigger differences between the highest and lowest point of about 40 nm versus 15 nm for the control **d,** Septal architecture of SH1000 and Δ*divIC* grown for 3 h without IPTG. Square boxes (left panels) indicate septal areas that were magnified to high resolution (right panels). Blue circles highlight the presence of pores during septal development.

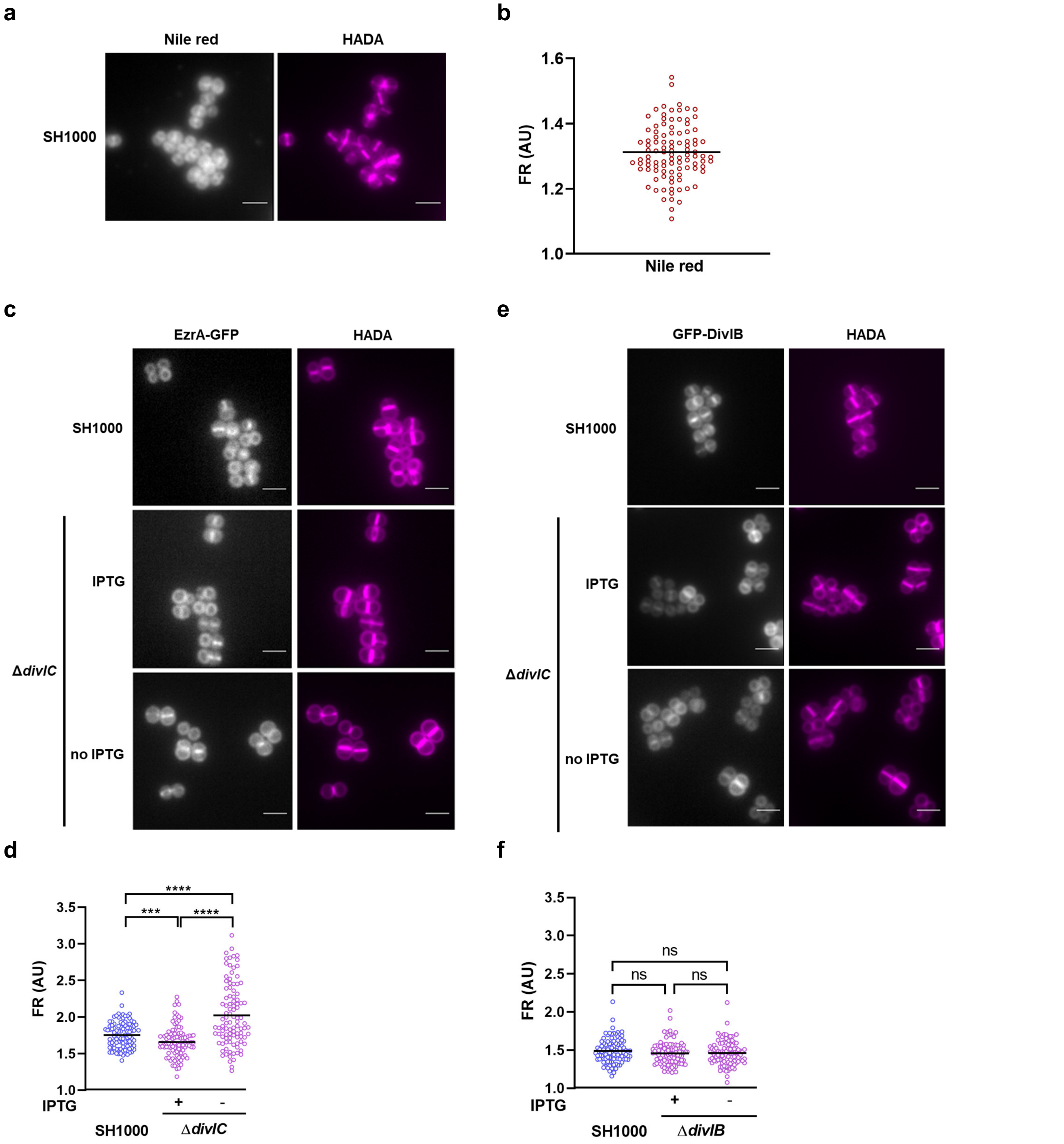

Supplementary figure 5. Role of DivIC in divisome protein localisation. **a,** SH1000 cells treated with Nile red to stain membranes and HADA to label PG used as a standard for FR values (see materials and methods). **b,** FR between Nile red fluorescent signal at the septum versus the cell periphery. **c,** Localisation of EzrA-GFP in SH1000 (VF104) and Δ*divIC* (SJF5558) cells grown with and without IPTG for 2 h and incubated with HADA for 30 min to label PG. **d,** FR between EzrA-GFP fluorescent signal at the septum versus the cell periphery. **e,** Localisation of GFP-DivIB in SH1000 (SJF5573) and Δ*divIC* (SJF5574) cells grown with and without IPTG for 2 h and incubated with HADA for 30 min to label PG. **f,** FR between GFP-DivIB fluorescent signal at the septum versus the cell periphery. Each circle indicates a single cell, lines are mean of an n= 100. *P* values were

determined by Mann–Whitney *U* tests (*** $P < 0.0005$, **** $P < 0.0001$). All images are average intensity projections of z stacks of cells with incomplete septa. Scale bars, 2 μm**.** Data is representative of two independent experiments.

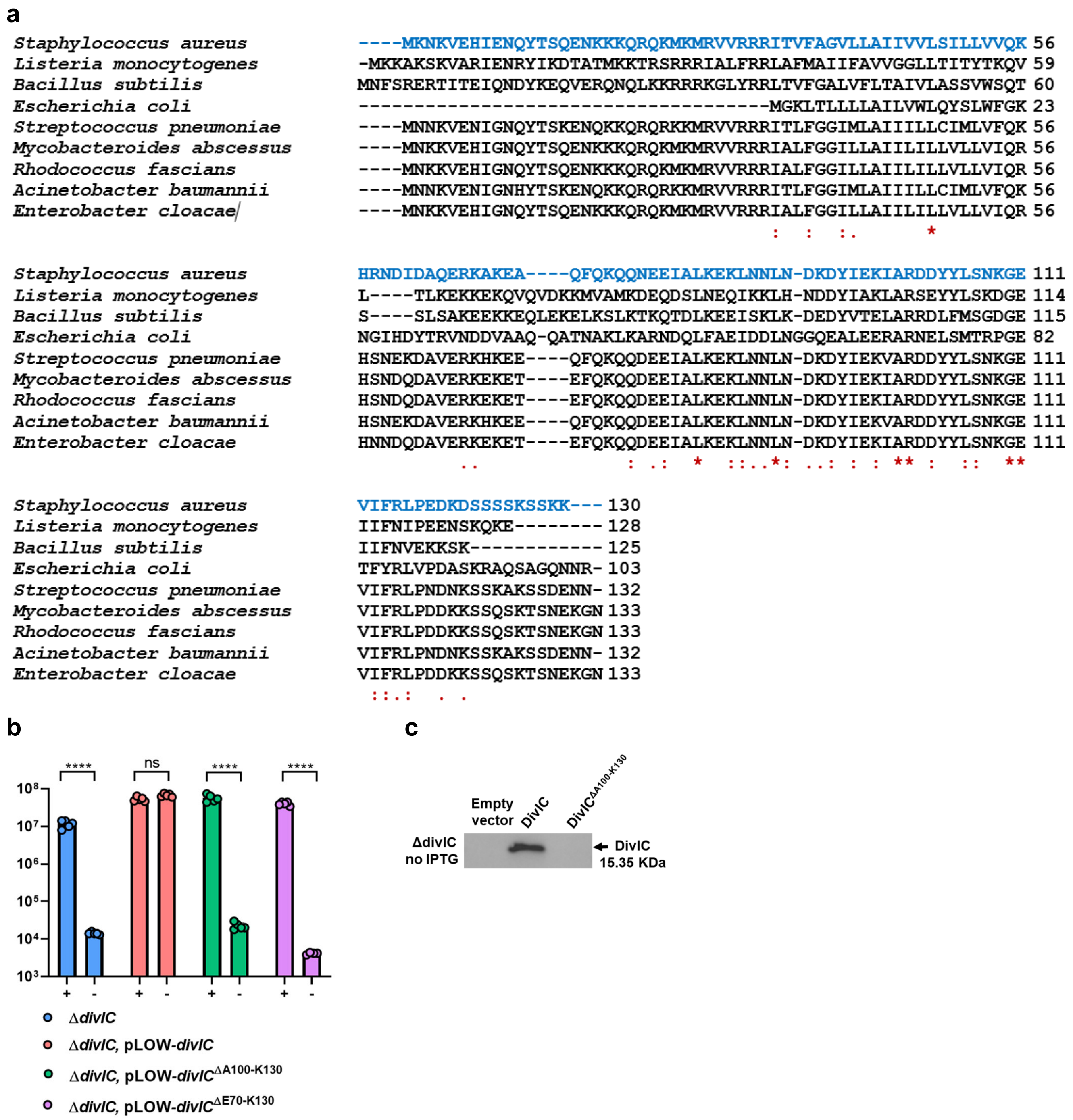

Supplementary figure 6. The importance and conservation of the exoplasmic domain of DivIC. a, Alignment of DiviC (FtsB) protein sequences. Asterisks indicate conserved residues; colons are conservative and full stops semi-conservative substitutions, respectively. *S. aureus* DivIC is highlighted in blue **b,** Viability of Δ*divIC* carrying the empty vector (SJF5544) or expressing DivIC (SJF5503), DivIC$^{\Delta A100\text{-}K130}$ (SJF5504) or DivIC$^{\Delta E70\text{-}K130}$ (SJF5505) in the presence and absence of IPTG. Data represents the mean and standard deviation of at least four independent experiments. *P* values were determined by two-tailed unpaired *t* test (**** *P* < 0.0001). **c,** Western blot of whole cell lysates from Δ*divIC* carrying the empty vector or expressing DivIC and DivIC$^{\Delta A100\text{-}K130}$. Cultures were grown without IPTG for 2 h. Anti-DivIC antibodies were used for detection.

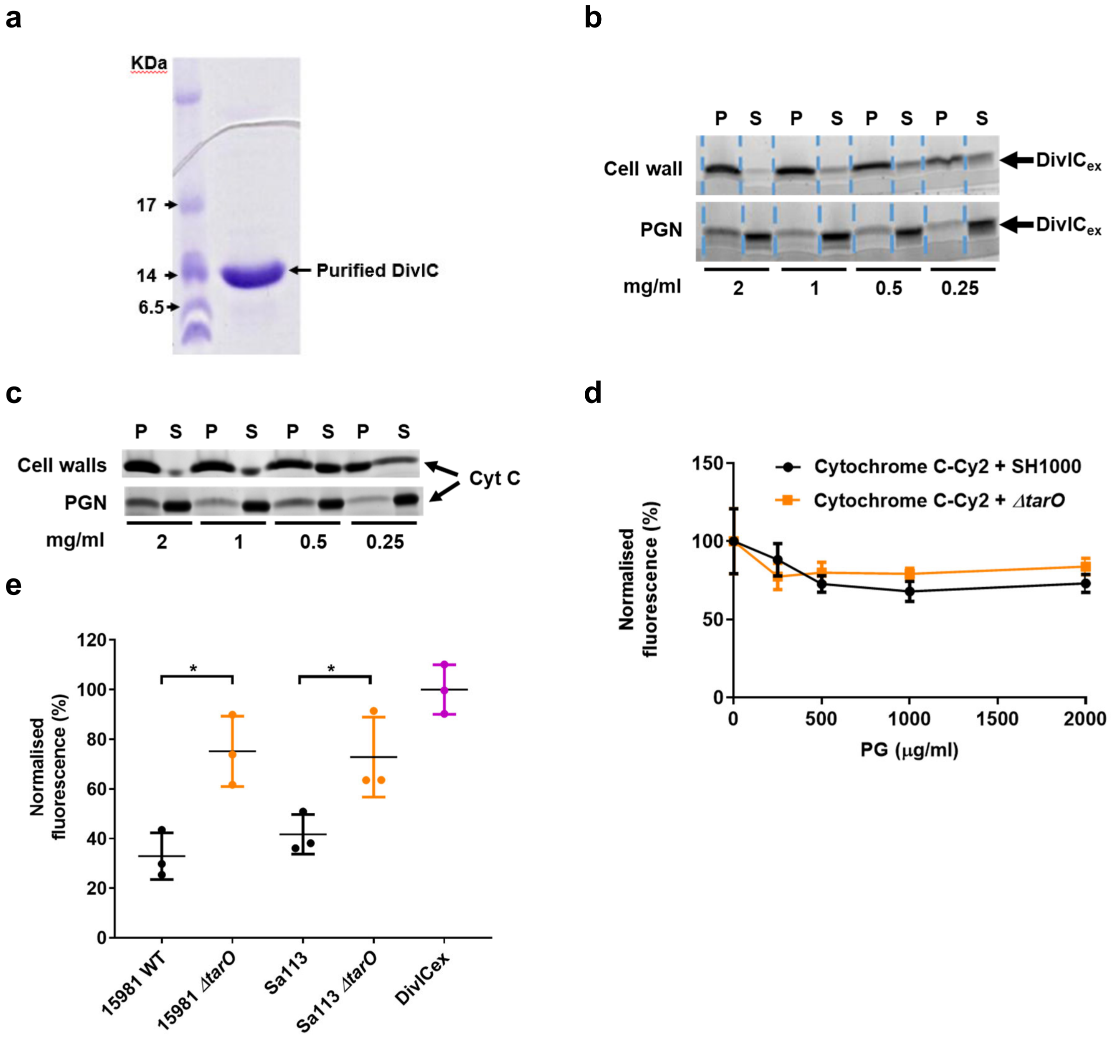

Supplementary figure 7. Binding of recombinant DivIC to cell wall material. a, Purified recombinant DivIC. **b,** Binding of the recombinant exoplasmic domain of DivIC (DivIC$_{ex}$) to purified cell walls or PG from SH1000. After incubation at room temperature for 5 min, insoluble pellet (P) and supernatant (S) fractions were separated by centrifugation and analysed by SDS-PAGE. **c,** Binding of recombinant cytochrome C (Cyt C) (0.1 mg/ml) to SH1000 cell walls and PG. P and S fractions were separated as in (b). **d,** Increasing concentrations of PG purified from SH1000 and Δ*tarO* (SJF5289) cells were incubated with Cy2-labeled Cyt C (500 nM) and unbound protein was measured in the supernatant after centrifugation. **e,** Cell walls (0.25 mg/ml) from 15981 and Sa113 *S. aureus* backgrounds and their respective Δ*tarO* mutants were incubated with Cyt2-labeled DivIC (500 nM) for 5 min and fluorescence measured in the supernatant after centrifugation. Mean and standard deviations of three repeats are shown. *P* values were calculated by two-tailed *t* test. (* *P* <0.05).

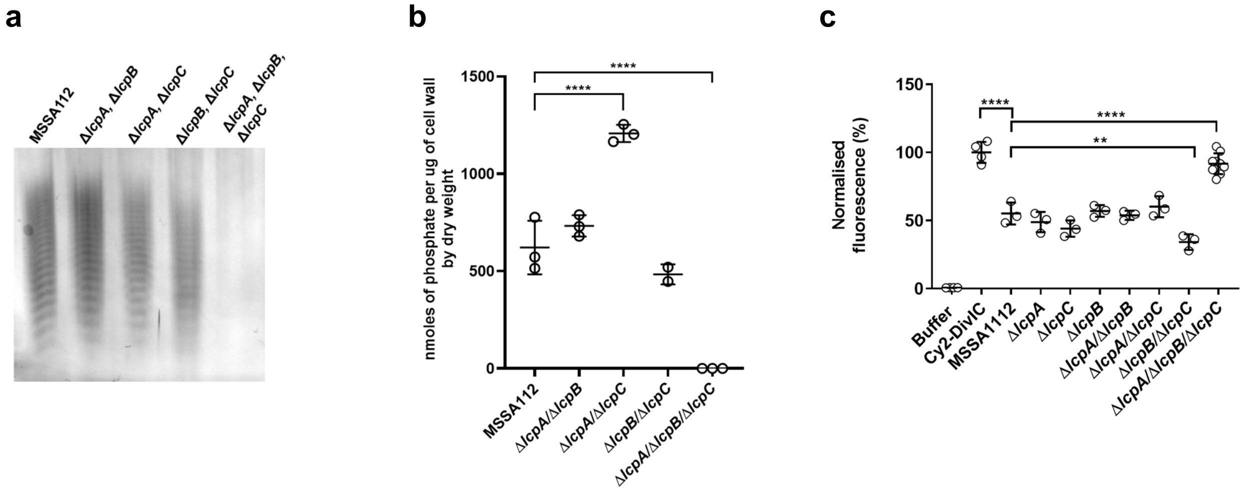

Supplementary figure 8. Role of the WTA ligases in DivIC binding to cell wall material. a, Cell walls were purified from MSSA112 (parental strain), Δ*lcpA,* Δ*lcpB* (RH72), Δ*lcpA,* Δ*lcpC* (PS60), Δ*lcpB,* Δ*lcpC* (PS109), Δ*lcpA,* Δ*lcpB*, Δ*lcpC* (PS111) and WTA isolated via alkaline hydrolysis. Purified WTA were visualised in a Native-PAGE stained with Alcian-silver. **b,** Phosphate content measured in isolated cell walls from MSSA112, and the *lcp* isogenic mutants. **c,** Cell walls (0.25 mg/ml) from MSSA112, Δ*lcpA* (JH100), Δ*lcpC* (PS47), Δ*lcpB* (RH53), Δ*lcpA,* Δ*lcpB* (RH72), Δ*lcpA,* Δ*lcpC* (PS60), Δ*lcpB,* Δ*lcpC* (PS109), Δ*lcpA,* Δ*lcpB*, Δ*lcpC* (PS111) were incubated with Cy2-labeled $DivIC_{ex}$ (500 nM) for 5 min and fluorescence measured in the supernatant after centrifugation. Circles represent three repeats. Bars are mean and standard deviations of three repeats. *P* values were determined by one way ANOVA & Dunnetts multiple comparisons (** *P* <0.01; **** *P* <0.001).

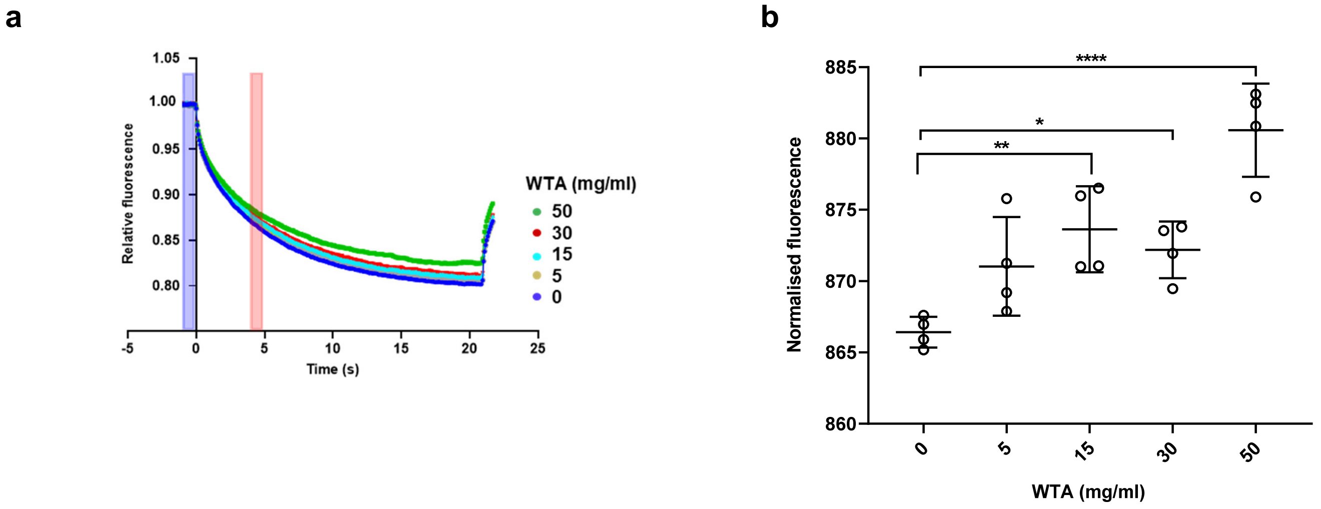

Supplementary figure 9. DivIC binds to WTA. a, Microscale thermophoresis analysis (MST) of AF647-labeled DivIC$_{ex}$ (100 nM) incubated with *S. aureus* WTA isolated from SH1000 cell walls. Traces shows the difference between change in detected fluorescence of DivIC-AF647 alone (blue) and DivIC-AF647 with increasing concentrations of WTA over time. The full trace displays the mean for four repeats. Purple shading shows the values prior to thermophoresis **b,** Mean and standard deviations of four repeats at 5 s (red vertical shading in (a)) are shown. *P* values were calculated by one-way ANOVA with Dunnett's multiple comparison (* P <0.05, ** P <0.01, **** P <0.0001).

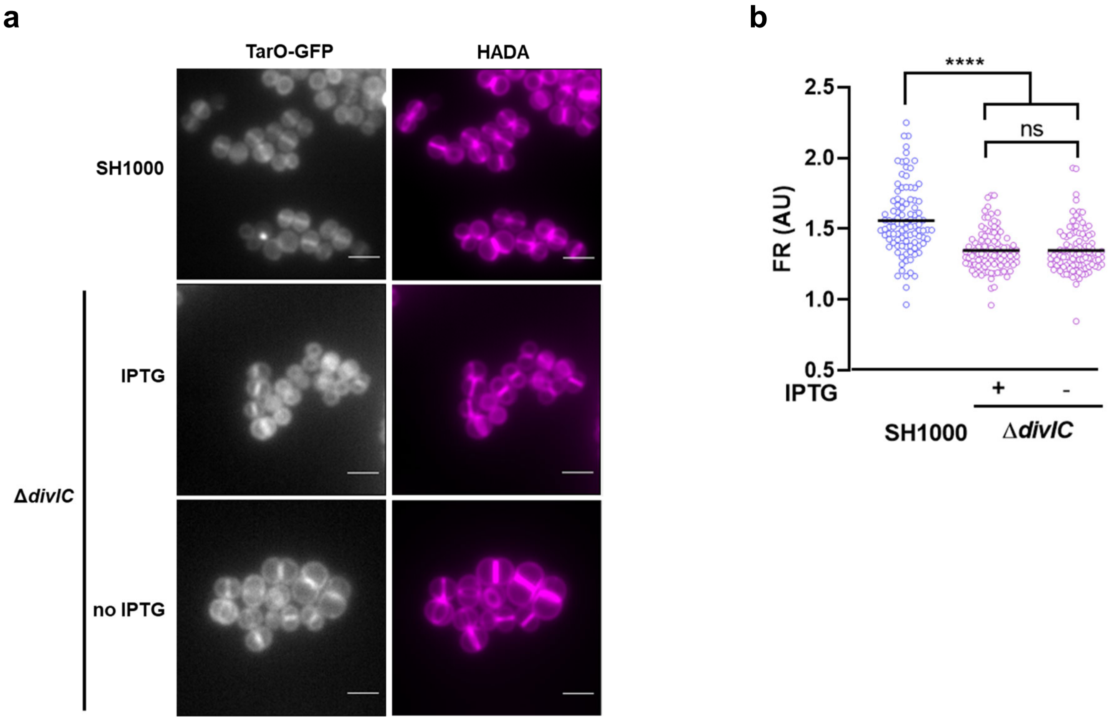

Supplementary figure 10. DivIC does not control TarO localisation. a, Localisation of TarO-GFP in SH1000 (SJF5507) and Δ*divIC* (SJF5508) cells grown with and without IPTG for 2 h and incubated with HADA for 30 min to label PG **b,** FR between TarO-GFP fluorescent signal at the septum versus the cell periphery. Each circle indicates a single cell, lines are mean of an n= 100. *P* values were determined by Mann–Whitney *U* tests (**** *P*< 0.0001). All images are average intensity projections of z stacks of cells with incomplete septa. Scale bar, 2 µm**.** Images are representative of two independent experiments.

Supplementary Table 1. Strains

Name	Relevant genotype/markers	Source
E. coli		
NEB5a		New England Biolabs
SJF3165	BL21(DE3) (Novagene), pALB26	This study
Staphylococcus aureus		
SH1000	Functional *rsbU*+ derivative of 8325–4	[1]
RN4220	Restriction deficient transformation recipient	[2]
CYL316	RN4220 pCL112Δ19; Cm^R	[3]
VF17	SH1000 pGL485 (*lacI*); Cm^R	[4]
SJF5450	SH1000 *geh*::P*spac~divIC*, *divIC*::*tet* (*lacI*); Kan^R, Tet^R, Cm^R	This study
SJF3883	SH1000 *spa*::*kan*, *geh*::P*spac~divIB*, Δ*divIB* (*lacI*); Kan^R, Tet^R, Cm^R	[5]
SJF5424	SH1000, pLOW-*gfp-divIC*; Ery^R	This study
SJF5427	SJF3883, pLOW-*gfp-divIC*; Tet^R, Cm^R, Ery^R	This study
SJF5541	VF17, pLOW-*gfp-pbp2*; Cm^R, Ery^R	This study
SJF5560	SJF5450, pLOW-*gfp-pbp2*; Kan^R, Tet^R, Cm^R, Ery^R	This study
SJF5768	VF17, pLOW-*ftsW-gfp*; Cm^R, Ery^R	This study
SJF5767	SJF5450, pLOW-*ftsW-gfp*; Kan^R, Tet^R, Cm^R, Ery^R	This study
VF104	VF17, *lysA*::ezrA-*gfp*; Cm^R, Ery^R	[4]
SJF5558	SJF5450, *lysA*::ezrA-*gfp*; Kan^R, Tet^R, Cm^R, Ery^R	This study
SJF5573	VF17, pLOW-*gfp-divIB*; Cm^R, Ery^R	This study
SJF5574	SJF5450, pLOW-*gfp-divIB*; Kan^R, Tet^R, Cm^R, Ery^R	This study
SJF5544	SJF5450, pLOW; Kan^R, Tet^R, Cm^R, Ery^R	This study
SJF5503	SJF5450, pLOW-*divIC*; Kan^R, Tet^R, Cm^R, Ery^R	This study
SJF5504	SJF5450, pLOW-*divIC*$^{\Delta A100\text{-}K130}$; Kan^R, Tet^R, Cm^R, Ery^R	This study
SJF5505	SJF5450, pLOW-*divIC*$^{\Delta E70\text{-}K130}$; Kan^R, Tet^R, Cm^R, Ery^R	This study
SJF5473	SJF5450, pLOW-*gfp-divIC*; Kan^R, Tet^R, Cm^R, Ery^R	This study
SJF5553	SJF5450, pLOW-*gfp-divIC*$^{\Delta A100\text{-}K130}$; Kan^R, Tet^R, Cm^R, Ery^R	This study
SJF5554	SJF5450, pLOW-*gfp-divIC*$^{\Delta E70\text{-}K130}$; Kan^R, Tet^R, Cm^R, Ery^R	This study
SJF5289	SH1000 Δ*tarO::ery*; Ery^R	[6]
SJF5396	pCU-*tarO*, Δ*tarO::ery*; Kan^R, Ery^R	[6]
15981	Clinical isolate	[7]
SJF3161	15981, Δ*tarO*	[8]
Sa113	Derivative of NCTC 8325	[9,10]
SJF2206	Sa113, Δ*tarO*	[11]
MSSA1112	Clinical isolate	[12]
PS47	MSSA1112 Δsa2103 (*lcpC*)	[13]
RH53	MSSA1112 Δ*sa0908 (lcpB)*	[13]
JH100	MSSA1112 Δ*msrR::ery (lcpA::ery*); Ery^R	[13]
RH72	MSSA1112 Δ*sa0908,* Δ*msrR::ery*; Ery^R	[13]
PS60	MSSA1112 Δ*sa2103,* Δ*msrR::ery*, Ery^R	[13]
PS109	MSSA1112 Δ*sa2103,* Δ*sa0908*, Ery^R	[13]
PS111	MSA1112 Δ*sa2103,* Δ*sa0908,* Δ*msrR ::ery* ; Ery^R	[13]
SJF5507	SH1000, pLOW-P*tarO-tarO-gfp* (*lacI);* Cm^R, Ery^R	This study
SJF5508	SJF5450, pLOW-P*tarO-tarO-gfp*; Kan^R, Tet^R, Cm^R, Ery^R	This study

SJF5398	SH1000, pCQ11-*gfp-divIC*; Kan^R	This study
SJF5395	SJF5289, pCQ11-*gfp-divIC*; Ery^R, Kan^R	This study

Amp^R, ampicillin resistant; Ery^R, erythromycin resistant; Tet^R, tetracycline resistant; Kan^R, kanamycin resistant; Cm^R, chloramphenicol resistant; $Spec^R$

Supplementary Table 2. Plasmids

Name	Relevant genotype/markers	Source
pALB26	pET21d with an N-terminal 6xHis tag ligated to the DivIC extracellular fragment (K56 to K130); Amp^R	This study
pKASBAR	pUC18 containing *attP* and a kanamycin resistance cassette (Amp^R, Kan^R)	[5]
pMAD	*E. coli-S. aureus* shuttle vector with temperature-sensitive origin of replication in *S. aureus* and promoterless *bgaB*; Amp^R, Ery^R	[14]
pKASBAR-*divIC*	pKASBAR-kan, Pspac-divIC; Amp^R, Kan^R	Genewiz UK Ltd
pMAD-U-D*divIC*	pMAD carrying 904 bp upstream and 960 bp downstream *divIC* with a NotI site between the up and downstream sequences; Ery^R	This study
pOB-tet	pGEM3Zf(+) cloning vector containing the tetracycline resistance cassette from pAISH; Amp^R, Kan^R, Tet^R	[15]
pMAD-U-*tet*-D*divIC*	pMAD carrying 904 bp upstream and 960 bp downstream divIC with a tetracycline cassette between the up and downstream sequences; Ery^R, Tet^R	This study
pLOW	pSK41-type low copy number plasmid; Amp^R, Ery^R	[16]
pLOW-*gfp-pbp2*	pLOW expressing a *gfp-pbp2* fusion under control of the penicillinase constitutive promoter (P*pcn*); Ery^R	Genwiz UK Ltd
pLOW-*ftsW-gfp*	pLOW expressing a *ftsW-gfp* fusion under control of the penicillinase constitutive promoter (P*pcn*); Ery^R	This work
pLOW-*gfp-divIB*	pLOW expressing a *gfp-divIB* fusion under control of the penicillinase constitutive promoter (P*pcn*); Ery^R	Genwiz UK Ltd
pCQ11	*E. coli-S. aureus* shuttle vector containing *lacI*; Amp^R, Ery^R	[17]
pCQ11-*gfp-divIC*	pCQ11 expressing *gfp-divIC* under control of the penicillinase constitutive promoter (P*pcn*); Ery^R	Genewiz UK Ltd
pCQ11-*divIC*	pCQ11 expressing *divIC* under control of the penicillinase constitutive promoter (P*pcn*); Ery^R	This study
pLOW-*divIC*	pLOW expressing *divIC* under control of the penicillinase constitutive promoter (P*pcn*); Ery^R	This study
pLOW-*divIC*$^{\Delta A100\text{-}K130}$	pLOW expressing *divIC*$^{\Delta A100\text{-}K130}$ under control of the penicillinase constitutive promoter (P*pcn*); Ery^R	This study
pLOW-*divIC* $^{\Delta E70\text{-}K130}$	pLOW expressing *divIC* $^{\Delta E70\text{-}K130}$ under control of the penicillinase constitutive promoter (P*pcn*); Ery^R	This study
pLOW-*gfp-divIC*	pLOW expressing *gfp-divIC* under control of the penicillinase constitutive promoter (P*pcn*); Ery^R or Kan^R	This study
pLOW-*gfp-divIC*$^{\Delta A100\text{-}K130}$	pLOW expressing gfp-*divIC*$^{\Delta A100\text{-}K130}$ under control of the penicillinase constitutive promoter (P*pcn*); Ery^R	This study
pLOW-*divIC* $^{\Delta E70\text{-}K130}$	pLOW expressing gfp-*divIC* $^{\Delta E70\text{-}K130}$ under control of the penicillinase constitutive promoter (P*pcn*); Ery^R	This study

pLOW-*tarO-gfp*	pLOW expressing *tarO-gfp* under control of the *tarO* native promoter Ery^R	Genwiz UK Ltd

Amp^R, ampicillin resistant; Ery^R, erythromycin resistant; Tet^R, tetracycline resistant; Kan^R, kanamycin resistant; Cm^R, chloramphenicol resistant.

Supplementary table 3. Oligonucleotides

**Oligonucleotide name and restriction site**	**Sequence (5' to 3')***	**Application**	**Source**
MT55 U*divIC*Fw BglII	ataataAGATCTattgtcactggtg cacgaag	Amplification of region upstream of *S. aureus divIC*. Forward primer	This study
AK22 U*divIC*Rev NotI	ataataGCGGCCGCcctccaat ttacgctt	Amplification of region upstream of *S. aureus divIC*. Reverse primer	This study
AK23 U*divIC*Fw NotI	ataata<u>GCGGCCGC</u>aggatttat ttaacatatgtcaa	Amplification of region downstream of *S. aureus divIC* Forward primer	This study
MT56 D*divIC*Rv EcoRI	ataataGAATTCGtccaaatgat gcgccgttag	Amplification of region downstream of *S. aureus divIC*. Reverse primer	This study
MT57Fw NotI	ataataGCGGCCGCggaggag aagcatctgcagatag	Amplification of the tetracycline cassette from pOB. Forward primer	This study
MT58Rv NotI	ataataGCGGCCGCctctccca aagttgatccctta	Amplification of the tetracycline cassette from pOB. Reverse primer	This study
MT80Fw	atgaaaaataaagtagaacatatag aaaatc	For deletion of *gfp* from pCQ11-*gfp-divIC* by site directed mutagenesis	This study
MT81Rv	tgcttgtcacctccaataaatc	For deletion of gfp from pCQ11-*gfp*-divIC by site directed mutagenesis	This study
MT87Fw	tagcatgtctcattcaattccgcaacg caattaatgtg	To amplify P*pcn-divIC* and P*pcn*-gfp-*divIC* from pCQ11-*divIC* and pCQ11-*gfp-divIC* respectively	This study
MT88Rv	cgaaaagtgccacctgacgtttattttt tcgaagattttgagc	To amplify P*pcn-divIC* and P*pcn-gfp-divIC* from pCQ11-*divIC* and pCQ11-gfp-*divIC* respectively	This study
MT89Rv	cgaaaagtgccacctgacgtttaaat tttttcaatgtaatctttgtc	To amplify P*pcn-divIC* $^{\Delta A100\text{-}K130}$ and P*pcn-gfp-divIC* $^{\Delta A100\text{-}K130}$ from pCQ11-*divIC* and pCQ11-gfp-*divIC* respectively	This study
MT90Rv	cgaaaagtgccacctgacgtttatttc gcttttcgctc	To amplify P*pcn-divIC* $^{\Delta E70\text{-}K130}$ and P*pcn-gfp-divIC* $^{\Delta E70\text{-}K130}$ from pCQ11-*divIC* and pCQ11-gfp-*divIC* respectively.	This study
ALB21Fw NcoI	ataataCCATGGcaaaaacatc gcaatgatattgat	Amplification of the extracellular domain of *S. aureus divIC*. Forward primer	This study
ALB22Rv XhoI	ataataCTCGAGttttttcgaagatt ttgagct	Amplification of the extracellular domain of *S. aureus divIC*. Reverse primer	This study

*Restriction sites are in capitals

Supplementary references

1 Horsburgh, M. J. *et al.* sigmaB modulates virulence determinant expression and stress resistance: characterization of a functional rsbU strain derived from *Staphylococcus aureus* 8325-4. *Journal of bacteriology* **184**, 5457-5467, doi:10.1128/jb.184.19.5457-5467.2002 (2002).

2 Kreiswirth, B. N. *et al.* The toxic shock syndrome exotoxin structural gene is not detectably transmitted by a prophage. *Nature* **305**, 709-712, doi:10.1038/305709a0 (1983).

3 Lee, C. Y., Buranen, S. L. & Ye, Z. H. Construction of single-copy integration vectors for *Staphylococcus aureus*. *Gene* **103**, 101-105 (1991).

4 Steele, V. R., Bottomley, A. L., Garcia-Lara, J., Kasturiarachchi, J. & Foster, S. J. Multiple essential roles for EzrA in cell division of *Staphylococcus aureus*. *Molecular microbiology* **80**, 542-555, doi:10.1111/j.1365-2958.2011.07591.x (2011).

5 Bottomley, A. L. *et al.* *Staphylococcus aureus* DivIB is a peptidoglycan-binding protein that is required for a morphological checkpoint in cell division. *Molecular microbiology*, doi:10.1111/mmi.12813 (2014).

6 Salamaga, B. *et al.* Demonstration of the role of cell wall homeostasis in *Staphylococcus aureus* growth and the action of bactericidal antibiotics. *Proceedings of the National Academy of Sciences of the United States of America* **118**, doi:10.1073/pnas.2106022118 (2021).

7 Valle, J. *et al.* SarA and not sigmaB is essential for biofilm development by *Staphylococcus aureus*. *Molecular microbiology* **48**, 1075-1087, doi:10.1046/j.1365-2958.2003.03493.x (2003).

8 Vergara-Irigaray, M. *et al.* Wall teichoic acids are dispensable for anchoring the PNAG exopolysaccharide to the *Staphylococcus aureus* cell surface. *Microbiology (Reading)* **154**, 865-877, doi:10.1099/mic.0.2007/013292-0 (2008).

9 Iordanescu, S. & Surdeanu, M. Two restriction and modification systems in *Staphylococcus aureus* NCTC8325. *Journal of general microbiology* **96**, 277-281, doi:10.1099/00221287-96-2-277 (1976).

10 Peschel, A. *et al.* Inactivation of the *dlt* operon in *Staphylococcus aureus* confers sensitivity to defensins, protegrins, and other antimicrobial peptides. *The Journal of biological chemistry* **274**, 8405-8410, doi:10.1074/jbc.274.13.8405 (1999).

11 Kohler, T., Weidenmaier, C. & Peschel, A. Wall teichoic acid protects *Staphylococcus aureus* against antimicrobial fatty acids from human skin. *Journal of bacteriology* **191**, 4482-4484, doi:10.1128/jb.00221-09 (2009).

12 Entenza, J. M., Vouillamoz, J., Glauser, M. P. & Moreillon, P. Levofloxacin versus ciprofloxacin, flucloxacillin, or vancomycin for treatment of experimental endocarditis due to methicillin-susceptible or -resistant *Staphylococcus aureus*. *Antimicrobial agents and chemotherapy* **41**, 1662-1667, doi:10.1128/aac.41.8.1662 (1997).

13 Over, B. *et al.* LytR-CpsA-Psr proteins in *Staphylococcus aureus* display partial functional redundancy and the deletion of all three severely impairs septum placement and cell separation. *FEMS microbiology letters* **320**, 142-151, doi:10.1111/j.1574-6968.2011.02303.x (2011).

14 Arnaud, M., Chastanet, A. & Débarbouillé, M. New vector for efficient allelic replacement in naturally nontransformable, low-GC-content, gram-positive bacteria. *Applied and environmental microbiology* **70**, 6887-6891, doi:10.1128/aem.70.11.6887-6891.2004 (2004).

15 Lund, V. A. *et al.* Molecular coordination of *Staphylococcus aureus* cell division. *eLife* **7**, doi:10.7554/eLife.32057 (2018).

16 Liew, A. T. F. *et al.* A simple plasmid-based system that allows rapid generation of tightly controlled gene expression in *Staphylococcus aureus*. *Microbiology (Reading)* **157**, 666-676, doi:10.1099/mic.0.045146-0 (2011).

17 Hardt, P. *et al.* The cell wall precursor lipid II acts as a molecular signal for the Ser/Thr kinase PknB of *Staphylococcus aureus*. *International journal of medical microbiology : IJMM* **307**, 1-10, doi:10.1016/j.ijmm.2016.12.001 (2017).
